# Drug information update. Lithium and chronic kidney disease: debates and dilemmas

**DOI:** 10.1192/pb.bp.116.054031

**Published:** 2017-08

**Authors:** Sumeet Gupta, Udayan Khastgir

**Affiliations:** 1 West Park Hospital, Darlington, UK

## Abstract

Lithium is an established treatment for bipolar disorder and an augmenting agent for treatment-resistant depression. Despite awareness of renal adverse effects, including chronic kidney disease, for the past five decades, there has been a lack of research evidence. This has led to debates around the existence and magnitude of the risk. This article discusses the current evidence base regarding the link between lithium and chronic kidney disease, monitoring of renal functions and its clinical implications.

Lithium is one of the oldest psychotropic medications. It remains the gold standard treatment for bipolar disorder and an augmenting agent for treating depression. Over the past few decades, additional benefits of lithium have also come to light, most importantly its role in preventing suicide and Alzheimer's disease.^1,2^ As it is often used as a maintenance treatment for lifelong disorders, concerns have been raised about its potential long-term organ toxicity, mainly the effect on glomerular function leading to chronic kidney disease (CKD).^3–5^

## Debate 1: does lithium cause CKD?

The association between CKD and lithium has been known for a long time, and monitoring renal function in patients receiving lithium therapy has been the norm for many decades. Despite this, there has been little research into the renal adverse effects of lithium so far. A link between lithium therapy and CKD has received increasing recognition, and there are several explanations for this. First, glomerular function decreases gradually as a normal part of the ageing process; increased life expectancy has led to increasing numbers of lithium patients being diagnosed with CKD. Second, there has been a resurgence of interest in lithium and its safe use, owing to its proven effectiveness in bipolar disorder in comparison with other mood stabiliser drugs and its anti-suicide effect. Last, more effective monitoring of renal function and the use of more sensitive measures to diagnose CKD, such as the estimated glomerular filtration rate (eGFR) rather than serum urea and creatinine levels, have led to the early identification of affected patients.

Despite significant progress over the past two decades, doubts still remain about the existence and magnitude of the risk.^3–5^ Unlike its tubular adverse effects, which more commonly present with polyuria and polydipsia, the glomerular adverse effects of lithium therapy appear after long-term use and might not normalise or improve after its discontinuation. It has therefore been difficult to attribute causality for this adverse effect.

The debate about lithium nephrotoxicity started in 1977, with reported abnormalities in renal biopsies from a small group of patients treated with lithium.^6^ Initial cross-sectional, case-control and small cohort studies with short-term follow-ups reported contradictory findings on the toxicity risk.^7–10^ In 1994, Bendz *et al* published a study of 142 patients who had been taking lithium for more than 15 years. The authors measured the glomerular filtration rate (GFR) by chromium-51-labelled ethylene diamine tetra-acetic acid ([^51^Cr]EDTA) clearance and found that in 21% of patients the GFR was lower than in the demographic-matched population control group.^11^ Two meta-analyses of these diverse studies failed to produce conclusive evidence for or against lithium nephrotoxicity. Although they showed that patients on lithium therapy had worse renal function than controls (as measured by serum creatinine levels or the eGFR), they reported that the clinical significance was questionable. Both meta-analyses highlighted poor quality data and significant heterogeneity among studies.^12,13^

Recent epidemiological studies utilising large databases have consistently shown that CKD (defined as an eGFR of < 60 ml/min/1.73 m^2^) is common among lithium patients but that lithium therapy increases the risk of CKD. In addition, renal failure in patients with end-stage renal disease (ESRD) is not as rare as previously thought.^14–17^ In a retrospective cohort study using the General Practice Research Database, Close *et al* reported that patients taking lithium for bipolar disorder had a twofold increased risk of developing renal failure and a two-and-a-half-fold increased risk of developing CKD of any stage compared with lithium-naive patients.^14^

The relationship between lithium therapy and renal failure or ESRD has been investigated in many other studies that mainly use data from databases of patients undergoing renal replacement therapy (dialysis or renal transplant). A Swedish study found that 0.81% of renal replacement therapy patients had kidney disease attributable to lithium-induced nephropathy. Moreover, the risk of ESRD in lithium-treated patients was nearly sixfold greater than in the general population.^17^ Another recent retrospective cohort study, of renal replacement therapy patients in Australia, reported that the incidence rate of ESRD due to lithium therapy had increased significantly from 0.14 cases per million population per year (95% CI 0.06–0.22) in 1992–1996 to 0.78 cases per million population per year (95% CI 0.67–0.90) in 2007–2011. This report emphasised that lithium-induced nephropathy is not a rare cause of ESRD, and is becoming more common.^18^ However, the debate is far from over and contradictory findings continue to be published. For example, in a review of patients with ESRD in Sweden, Aiff *et al* found 32 cases of ESRD in patients who had started taking lithium before 1980 but none in those who had started taking lithium after 1980. Hence, the authors suggested that the opposite was true: renal failure might not be a problem with the current practice of maintaining lower serum lithium levels along with better monitoring of renal function.^19^ In a recent population-based retrospective cohort study in Denmark, Kessing *et al* also failed to identify any patients with lithium-induced ESRD. However, their findings indicated that bipolar disorder is independently associated with CKD.^20^ Therefore, uncertainties about the magnitude of the CKD risk are yet to be resolved.

It is clear that not all patients taking lithium experience glomerular adverse effects. Most likely, only a select group of patients develop CKD and only a small proportion of these progress to ESRD.^21^ Moreover, the association between lithium therapy and CKD is unlikely to be entirely explained by coincidence or confounding variables (such as age, other psychotropic drugs or comorbid medical/psychiatric illnesses). Discrepancies among studies were largely due to methodological differences such as varied parameters to assess renal function and definitions of renal impairment, short-term follow-up, a lack of patients on long-term lithium therapy, combining high-risk and low-risk groups, choice of control group (healthy *v*. psychiatric patients), and an inability to control the confounding variables. Definitive data on the magnitude of the risk are still lacking. Moreover, in the absence of any pathognomonic histological or biochemical changes, lithium-associated CKD remains a diagnosis of exclusion.

## Debate 2: is there any relationship between the tubular and glomerular adverse effects of lithium?

It was previously assumed that CKD is preceded by tubular adverse effects of lithium. However, differences in the prevalence of tubular and glomerular adverse effects and a lack of correlation between reduced glomerular function and tubular abnormalities on renal biopsy (in the form of tubular dilation and microcysts) argue against this assumption.^22,23^ Despite this, the presence of tubular adverse effects is suggested to increase the risk of CKD, hence the suggestion that treating or preventing tubular adverse effects might help to prevent deterioration of glomerular function.^21^

## Debate 3: is it possible to identify patients at high risk of developing CKD/ESRD and to predict the prognosis of these adverse effects?

As only a small proportion of patients on lithium therapy experience CKD, early identification of these high-risk patients might help to prevent and manage this adverse effect. Over the years, researchers have tried to identify both susceptibility and treatment-related factors such as associations with ageing, comorbid physical/psychiatric health problems, cumulative lithium dose or treatment duration, dosing frequency, and number of toxicity episodes.

Acute lithium toxicity is known to cause acute renal failure, and many patients suffer renal impairment even after recovering from an acute episode. There is consistent evidence that acute nephrotoxicity episodes can lead to CKD.^6,9,24^ Recently, Clos *et al* suggested that lithium-associated CKD is primarily mediated by acute lithium toxicity, and that avoiding lithium toxicity can prevent renal impairment^25^ Studies suggest a relationship between impaired renal function and either persistent high serum lithium levels (>0.6mmol/L *v*. <0.6mmol/L) or a single serum lithium measure of > 1.0 mmol/L. As these were not prospective studies, it is difficult to establish a causal relationship, especially as reduced renal function can also increase serum lithium levels.^26,27^ On the other hand, a recent randomised placebo controlled trial of low-dose lithium therapy (serum lithium levels of 0.25–0.50 mmol/L) in elderly patients with mild cognitive impairment did not show a significant difference in eGFR over a 4-year follow-up period.^28^ The study suggests that lower therapeutic levels of lithium might not impair renal function.^28^ In contrast, other studies have failed to show a relationship between CKD risk and lithium dose or serum lithium levels.^17,24^ It is well established that higher serum lithium levels provide better protection against another affective episode (especially a manic episode). Therefore, the debate about what serum lithium level represents a balance between safety and effectiveness is likely to continue until more definitive data become available.

Once-daily dose is thought to be associated with less renal impairment than multiple daily doses.^21^ Although a few studies do not support this, none have so far reported disadvantages for once-daily dosing.^29^ Therefore, although a definitive answer is lacking, it makes sense to adopt a once-daily dosing strategy. Comorbid physical health conditions such as diabetes or hypertension can independently cause CKD, but CKD is also commonly seen in lithium-treated patients, even in the absence of a comorbid physical illness. Therefore, comorbidity is unlikely to entirely explain the association between lithium and CKD.

So far, evidence about CKD risk factors is limited and somewhat contradictory. However, it indicates that lower therapeutic lithium doses might have a reduced detrimental effect on renal function and that acute lithium toxicity should be avoided to prevent renal dysfunction. Furthermore, once-daily dosing might be safer than multiple daily doses.

## Debate 4: how can we effectively monitor glomerular adverse effects?

Monitoring renal function in patients on lithium therapy has been the norm for many years, but there are significant discrepancies among the different guidelines on the parameters that should be used and the frequency of monitoring.^30–32^ The UK National Institute for Health and Care Excellence (NICE) recommends 6-monthly monitoring; the British Association for Psychopharmacology recommends annual monitoring; and the American Psychiatric Association recommends monitoring every 2–3 months for the first 6 months, followed by 6-monthly to annual measurements ^30–32^ However, they do not give specific guidance about the parameters for measuring renal function and continue to recommend measuring serum urea and creatinine levels, although recent guidelines have started to recommend measuring the eGFR. In other medical areas, measuring serum urea and creatinine levels is no longer a preferred option for monitoring renal function: standard practice is to monitor the eGFR. Many equations can be used to calculate the eGFR from serum creatinine concentration, with differing accuracies. Recent NICE guidelines on managing CKD suggest using the CKD Epidemiology Collaboration (CKD-EPI) equation. They also suggest using the CKD-EPI equation based on cystatin C levels if accurate GFR estimates are necessary.^33^ Psychiatry guidelines do not recommend any particular method for calculating the eGFR.

The importance of measuring proteinuria to monitor renal function in CKD patients is now firmly established. Proteinuria is an independent predictor of CKD progression, cardiovascular disorders and all-cause mortality.^33^ However, a role for estimating and monitoring proteinuria in lithium-related renal impairment is yet to be established. The evidence so far is sparse and contradictory: some reports suggest that proteinuria is linked to lithium-associated CKD and indicates a poor prognosis, while others suggest that lithium-associated CKD is not associated with proteinuria and that in the presence of proteinuria one should rule out other causes.^5,10,21^ However, recent publications have highlighted the importance of monitoring proteinuria in patients with lithium-associated CKD.^21,34^

Unfortunately, psychiatric guidelines have not kept up with advances in nephrology, and we need guidelines for evidence-based monitoring of renal function. Recent publications suggest that renal function should be monitored regularly via the eGFR and that the degree of proteinuria should be measured in patients with a declining eGFR or an eGFR of <60 ml/min/1.73 m.^2,21,34^ However, these recommendations are developed for CKD associated with other aetiological factors, and more specific evidence-based monitoring guidelines need to be developed to screen and monitor lithium-associated CKD.

## Dilemma 1: should lithium be stopped if a patient develops CKD?

The most common dilemma clinicians face is what to do if a patient on lithium therapy develops CKD. In this scenario, the clinician must decide whether to continue or discontinue lithium. Advice in the literature is contradictory, ranging from discontinuing lithium as soon as renal function starts to deteriorate (as evidenced by two consecutive tests) to continuing lithium even in the presence of CKD.^18,35^ The decision to discontinue lithium is based on the assumption that lithium is a causative factor for CKD and that its discontinuation would improve renal function or at least slow down deterioration. However, neither of these assumptions is completely supported by current evidence.^4,5,36^ Moreover, we still do not know whether lithium-associated CKD is reversible or irreversible. It has been suggested that this adverse effect might be reversible at the initial stages, only becoming irreversible after a certain threshold is reached.^4,21^ Presne *et al* suggested that the threshold might be somewhere between a GFR of 25 and 40 ml/min/1.73 m^2^.^22^ Thus, the advantages of discontinuing lithium are uncertain ^4,5,35,36^ On the other hand, there is enough evidence to suggest that lithium discontinuation is associated with high risk of relapse for patients with mood disorders, especially those with bipolar disorder. Moreover, the illness might become treatment refractory.^21^ One of the advantages of lithium is its anti-suicide effect: the risk of suicide is known to increase after lithium discontinuation^37^ The decision to continue or discontinue lithium treatment should thus only be taken after careful assessment of the benefits and risks, and because of uncertainties surrounding these, it is essential that the decision-making process should include patients and all of the professionals involved, including nephrologists. Our experience agrees with a documented report that many psychiatric patients prefer to maintain their mental stability against the unknown risk of further deterioration in renal function.^38^ In clinical practice, it is not unusual to request that a nephrologist makes this treatment decision. However, it is important that psychiatrists should not abdicate responsibility, because nephrologists might not be fully aware of the risks associated with the psychiatric illness.^5,21,34,35^ Another option would be to continue lithium treatment while closely monitoring renal function. Many authors have suggested trying to keep the lithium level at the lower end of the therapeutic range, although there is not much evidence that this prevents further deterioration in renal function. However, as CKD patients are particularly prone to lithium toxicity, this strategy appears prudent.

## Dilemma 2: should we consider lithium therapy for patients already diagnosed with CKD?

There is not much research evidence to support or dispute this decision. Lithium treatment may lead to further deterioration in renal function, which could be clinically important because the renal reserve is already low in patients with CKD. A study of elderly patients suggested that individuals with pre-existing CKD were more susceptible to a lithium-associated decline in renal function^36^ On the other hand, we should not deprive such patients of an effective therapy because of unproven adverse consequences. In 2012, Werneke *et al* designed a mathematical model based on the existing, but limited, evidence to analyse the risks and benefits of continuing or discontinuing lithium therapy for CKD patients. They concluded that most patients should continue lithium treatment even if long-term renal adverse effects develop. They also recommended prescribing lithium to CKD patients because treatment benefits outweighed the risks.^35^ However, at present there is not enough evidence to support any decision.

## Conclusions

Limited knowledge of its renal (especially glomerular) adverse effects has led clinicians to either avoid or prematurely discontinue lithium therapy because of the perceived risk of a negative renal outcome. Over the past decade, a few large database studies have confirmed the existence of lithium-associated CKD, but uncertainty remains about the magnitude and determinants of the risks. Lithium therapy is here to stay and we should learn to optimise its efficacy and safety. There is a need for large-scale prospective studies focused on the early identification of high-risk patients and for developing evidence-based guidelines to monitor renal function in patients treated with lithium.

## References

[1] Cipriani A Hawton K Stockton S Geddes JR Lithium in the prevention of suicide in mood disorders: updated systematic review and meta-analysis. BMJ 2013; 346: f3646. 23814104 10.1136/bmj.f3646

[2] Young AH More good news about the magic ion: lithium may prevent dementia, Br J Psychiatry 2011; 198: 336–7. 21525515 10.1192/bjp.bp.110.082875

[3] Gitlin M Lithium and the kidney: an updated review. Drug Saf 1999; 20: 231–43. 10221853 10.2165/00002018-199920030-00004

[4] Raja M Lithium and kidney, 60 years later. Curr Drug Saf 2011; 6: 291–303. 22424536 10.2174/157488611798918737

[5] Adam WR Schweitzer I Walker RG Trade-off between the benefits of lithium treatment and the risk of chronic kidney disease. Nephrology 2012; 17: 776–9. 23107125 10.1111/j.1440-1797.2012.01641.x

[6] Hestbech J Hansen HE Amdisen A Olsen S Chronic renal lesions following long-term treatment with lithium. Kidney Int 1977; 12: 205–13. 926612 10.1038/ki.1977.102

[7] Jorkasky DK Amsterdam JD Oler J Braden G Alvis R Geheb M Lithium induced renal disease: a prospective study. Clin Nephrol 1988; 30: 293–302. 3243040

[8] Povlsen UJ Hetmar O Ladefoged J Bolwig TG Kidney functioning during lithium treatment: a prospective study of patients treated with lithium for up to ten years. Acta Psychiatr Scand 1992; 85: 56–60. 1546549 10.1111/j.1600-0447.1992.tb01442.x

[9] Hetmar O Povlsen UJ Ladefoged J Bolwig TG Lithium: long-term effects on the kidney. A prospective follow-up study ten years after kidney biopsy, fir J Psychiatry 1991; 158: 53–8. 10.1192/bjp.158.1.53 1901749

[10] Walker RG Davies BM Holwill BJ Dowling JP Kincaid-Smith P A clinico-pathological study of lithium nephrotoxicity. J Chronic Dis 1982; 35: 685–95. 7096532 10.1016/0021-9681(82)90021-2

[11] Bendz H Aurell M Balldin J Mathé AA Sjödin I Kidney damage in long-term lithium patients: a cross-sectional study of patients with 15 years or more on lithium. Nephrol Dial Transplant 1994; 9: 1250–4. 7816284

[12] Paul R Minay J Cardwell C Fogarty D Kelly C Meta-analysis of the effects of lithium usage on serum creatinine levels. J Psychopharmacol 2010; 24: 1425–31. 19395432 10.1177/0269881109104930

[13] Mcknight RF Adida M Budge K Stockton S Goodwin GM Geddes JR Lithium toxicity profile: a systematic review and meta-analysis. Lancet 2012; 379: 721–8. 22265699 10.1016/S0140-6736(11)61516-X

[14] Close H Reilly J Mason JM Kripalani M Wilson D Main J Renal failure in lithium treated bipolar disorder: a retrospective cohort study. PLoS ONE 2014; 9: e90169. 24670976 10.1371/journal.pone.0090169 PMC3966731

[15] Shine B McKnight RF Leaver L Geddes JR Long-term effects of lithium on renal, thyroid, and parathyroid function: a retrospective analysis of laboratory data. Lancet 2015; 386: 461–8. 26003379 10.1016/S0140-6736(14)61842-0

[16] Bocchetta A Ardau R Carta P Ligas F Sardu C Pani A Duration of lithium treatment is a risk factor for reduced glomerular function: a cross-sectional study. BMC Med 2013; 11: 33. 23399351 10.1186/1741-7015-11-33 PMC3606463

[17] Bendz H Schön S Attman PO Aurell M Renal failure occurs in chronic lithium treatment but is uncommon. Kidney Int 2010; 77: 219–24. 19940841 10.1038/ki.2009.433

[18] Roxanas M Grace BS George CRP Renal replacement therapy associated with lithium nephrotoxicity in Australia. Med J Aust 2014; 200: 226–8. 24580527 10.5694/mja13.10435

[19] Aiff H Attman PO Aurell M Bendz H Schön S Svedlund J The impact of modern treatment principles may have eliminated lithium-induced renal failure. J Psychopharmacol 2014; 28: 151–4. 24346809 10.1177/0269881113516202

[20] Kessing LV Gerds TA Feldt-Rasmussen B Andersen PK Licht RW Use of lithium and anticonvulsants and the rate of chronic kidney disease: a nationwide population-based study. JAMA Psychiatry 2015; 72: 1182–91. 26535805 10.1001/jamapsychiatry.2015.1834

[21] Gupta S Kripalani M Khastgir U Reilly J Management of the renal adverse effects of lithium. Adv Psychiatr Treat 2013; 19: 457–66.

[22] Presne C Fakhouri F Noel LH Stengel B Even C Kreis H Lithium-induced nephropathy: rate of progression and prognostic factors. Kidney Int 2003; 64: 585–92. 12846754 10.1046/j.1523-1755.2003.00096.x

[23] Rej S Elie D Mucsi I Looper KJ Segal M Chronic kidney disease in lithium-treated older adults: a review of epidemiology, mechanisms, and implications for the treatment of latelife mood disorders. Drugs Aging 2015; 32: 31–42. 25519823 10.1007/s40266-014-0234-9

[24] Lepkifker E Sverdlik A lancu I Ziv R Segev S Kotler M Renal insufficiency in long-term lithium treatment. J Clin Psychiatry 2004; 65: 850–6. 15291664 10.4088/jcp.v65n0618

[25] Clos S Rauchhaus P Severn A Cochrane L Donnan PT Long-term effect of lithium maintenance therapy on estimated glomerular filtration rate in patients with affective disorders: a population-based cohort study. Lancet Psychiatry 2015; 2: 1075–83. 26453408 10.1016/S2215-0366(15)00316-8

[26] Kirkham E Skinner J Anderson T Bazire S Twigg MJ Desborough JA One lithium level >1.0 mmol/l causes an acute decline in eGFR: findings from a retrospective analysis of a monitoring database. BMJ Open 2014; 4: e006020. 10.1136/bmjopen-2014-006020 PMC4225230 25380811

[27] Kehoe RF A cross-sectional study of glomerular function in 740 unselected lithium patients. Acta Psychiatr Scand 1994; 89: 68–71. 8140909 10.1111/j.1600-0447.1994.tb01488.x

[28] Aprahamian I Santos FS dos Santos B Talib L Diniz BS Radanovic M Long-term, low-dose lithium treatment does not impair renal function in the elderly: a 2-year randomized, placebo-controlled trial followed by single-blind extension. J Clin Psychiatry 2014; 75: e672–8. 25093483 10.4088/JCP.13m08741

[29] Carter L Zolezzi M Lewczyk A An updated review of the optimal lithium dosage regimen for renal protection. Can J Psychiatry 2013; 58: 595–600. 24165107 10.1177/070674371305801009

[30] American Psychiatric Association Practice guideline for the treatment of patients with bipolar disorder. Am J Psychiatry 2002; 159:1–50. 11958165

[31] National Institute for Health and Care Excellence *Bipolar Disorder (Update): The Management of Bipolar Disorder in Adults, Children and Adolescents in Primary and Secondary Care* (Clinical Guideline 185). NICE, 2014.

[32] Goodwin GM Consensus Group of the British Association for Psychopharmacology Evidence-based guidelines for treating bipolar disorder: revised second edition-recommendations from the British Association for Psychopharmacology. J Psychopharmacol 2009; 23: 346–88. 19329543 10.1177/0269881109102919

[33] National Institute for Health and Care Excellence *Chronic Kidney Disease in Adults: Assessment and Management* (Clinical Guideline 182). NICE, 2014. 32208570

[34] Kripalani M Shawcross J Reilly J Main J Lithium and chronic kidney disease. BMJ 2009; 339: b2452. 19578086 10.1136/bmj.b2452

[35] Werneke U Ott M Renberg ES Taylor D Stegmayr B A decision analysis of long-term lithium treatment and the risk of renal failure. Acta Psychiatr Scand 2012; 126:186–97. 22404233 10.1111/j.1600-0447.2012.01847.x PMC3440572

[36] Rej S Abitbol R Looper K Segal M Chronic renal failure in lithium-using geriatric patients: effects of lithium continuation versus discontinuation – a 60-month retrospective study. Int Geriatr Psychiatry 2013; 28: 450–3. 10.1002/gps.3841 22674617

[37] Baldessarini RJ Tondo L Viguera A Discontinuing lithium maintenance treatment in bipolar disorders: risks and implications. Bipolar Disord 1999; 1: 17–24. 11256650 10.1034/j.1399-5618.1999.10106.x

[38] Raedler TJ Wiedemann K Lithium-induced nephropathies. Psychopharmacol Bull 2007; 40: 134–49. 17514192

